# Increased susceptibility to collagen-induced arthritis in female mice carrying congenic *Cia40/Pregq2 *fragments

**DOI:** 10.1186/ar2470

**Published:** 2008-08-06

**Authors:** Maria Liljander, Åsa Andersson, Rikard Holmdahl, Ragnar Mattsson

**Affiliations:** 1 Lund Transgenic Core Facility, BMC C13, Lund University, Klinikgatan 28, SE-221 84 Lund, Sweden; 2 Department of Pharmacology and Pharmacotherapy, Group of Molecular Immunopharmacology, Faculty of Pharmaceutical Sciences, Copenhagen University, Universitetsparken 2, DK-2100 Copenhagen Ø, Denmark; 3 Medical Inflammation Research, Lund University, BMC I11, SE-221 84 Lund, Sweden; 4 Karolinska Institute, Division of Medical Inflammation Research, Sheeles väg 2, SE-171 77 Stockholm, Sweden

## Abstract

**Introduction:**

Collagen-induced arthritis (CIA) in mice is a commonly used experimental model for rheumatoid arthritis (RA). We have previously identified a significant quantitative trait locus denoted *Cia40 *on chromosome 11 that affects CIA in older female mice. This locus colocalizes with another locus, denoted *Pregq2*, known to affect reproductive success. The present study was performed to evaluate the role of the *Cia40 *locus in congenic B10.Q mice and to identify possible polymorphic candidate genes, which may also be relevant in the context of RA.

**Methods:**

Congenic B10.Q mice carrying an NFR/N fragment surrounding the *Cia40*/*Pregq2 *loci were created by 10 generations of backcrossing (N10). The congenic mice were investigated in the CIA model, and the incidence and severity of arthritis as well as the serum levels of anti-collagen II (CII) antibodies were recorded.

**Results:**

Significant effects on onset, incidence, severity, and anti-CII antibody titers were observed in female mice carrying a heterozygous congenic *Cia40/Pregq2 *fragment of NFR/N origin, containing one or more polymorphic genes. Congenic male mice did not show increased incidence of CIA, but males carrying a heterozygous fragment showed a significant increase in severity in comparison with wildtype B10.Q males (littermates).

**Conclusion:**

The *Cia40/Pregq2 *locus at chromosome 11 contains one or more polymorphic genes of NFR/N origin that significantly influence both incidence and severity of CIA in heterozygous congenic mice of the B10.Q strain. The major polymorphic candidate genes for the effects on CIA are *Cd79b*, *Abca8a*, and *Map2k6*. The congenic fragment also contains polymorphic genes that affect reproductive behavior and reproductive success. The *Sox9 *gene, known to influence sex reversal, is a candidate gene for the reproductive phenotype.

## Introduction

Collagen-induced arthritis (CIA) is a commonly used animal model for rheumatoid arthritis (RA). Although CIA shares several features with RA, there are some obvious differences between the mouse model and the human disease [1-3]. One such dissimilarity is the reversed sex susceptibility. A female predominance is characteristic for RA [4], whereas the opposite situation commonly is the case in mice developing CIA. Because of the male predominance of CIA in most strains of mice, including B10.Q, most published CIA experiments have been performed on males.

We have previously performed a genetic linkage analysis on multiparous female mice from an N2 cross between NFR/N and B10.Q, with the aim of finding CIA loci that are linked to disease development in females [5]. We identified one novel significant CIA-associated locus on chromosome 11, which is now denoted *Cia40*. No other CIA loci/genes have previously been found in this region, but the central part of chromosome 11 is known to contain a number of inflammation loci, such as *Eae22*, *Eae6b*, *Eae23*, and *Eae7 *[6-8]. However, none of the experimental autoimmune encephalitis (EAE) loci is located close to the *Cia40 *linkage peak, indicating that other polymorphic genes might be of importance.

Interestingly, in an additional quantitative trait locus (QTL) analysis with females of the same cross (N2 generation of NFR/N and B10.Q), we detected a highly significant QTL close to *Cia40 *on chromosome 11 linked to the trait 'pregnancy frequency' [9]. This locus is denoted *Pregq2 *and controls the frequency of successful pregnancies following successful copulation (successful coitus recorded by the detection of the 'vaginal plug'). In the initial QTL analysis, heterozygous mice carrying NFR/N genes at the *Pregq2 *locus suffered from an increased frequency of pregnancy failures [9]. We hypothesized that the *Cia40/Pregq2 *region of chromosome 11 may contain polymorphic genes that influence both CIA incidence and breeding success.

Although our original QTL analysis was performed on (aged) female mice with the hope of finding CIA loci with female predominance, there would still be a possibility that the *Cia40 *locus is of equal importance in both sexes. In the present paper, we present results indicating that *Cia40 *congenic females are more affected by CIA than males are. We also show that the *Cia40/Pregq2 *locus is linked to a disturbed reproductive behavior and reduced breeding performance in females.

## Materials and methods

### Mice

Inbred NFR/N mice were originally obtained from the National Institutes of Health (Bethesda, MD, USA) and the B10.Q mice were originally from the animal colony of Professor Jan Klein (Tübingen University, Tübingen Germany). (B10.Q × NFR/N) × B10.Q N_10 _mice were bred in the animal house of the Department of Pathology of Lund University, Sweden. The animals were fed standard rodent chow and water in a photoperiod of light/dark 12:12. All mice used in the present study had clean health monitoring protocols according to the recommendations of the Federation of European Laboratory Animal Sciences Association. The ethical permission for reproduction and arthritis (M236-06,) was provided by the Swedish Board of Agriculture.

### The *Cia40 *congenic mice and the fragment

**Figure 1:**
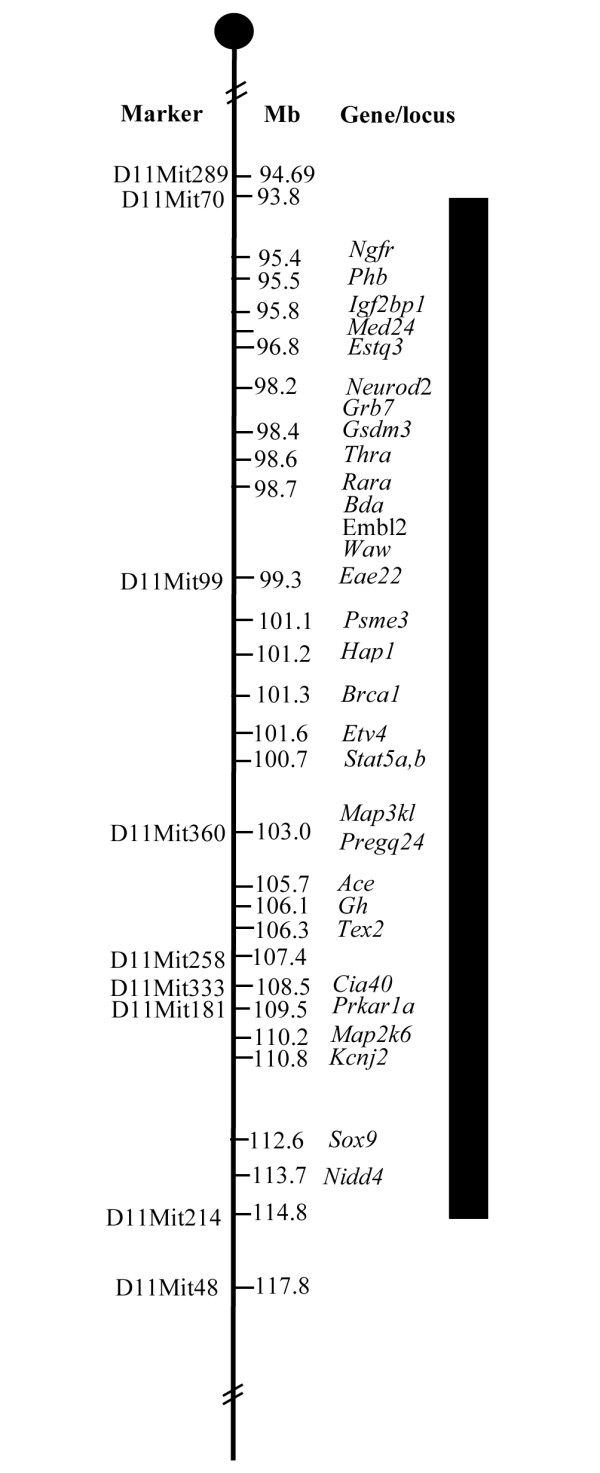
**Overview of the *Cia40/Pregq2 *congenic fragment**. The dark area indicates the genetic region from NFR/N in the congenic strain *Cia40/Pregq2*. The markers are placed according to Mouse Ensemble built 36 [18].

To confirm the previously identified linkage on chromosome 11, we backcrossed the NFR/N strain to the (more) CIA-resistant strain, B10.Q. Mice heterozygous for the congenic region (a small fragment from the NFR/N strain on B10.Q background) were chosen for additional backcrossing for 10 generations (Figure 1). All of the mice were derived from the same set of parents. Subsequently, the congenic mice were intercrossed. Mice heterozygous for NFR/N markers between D11Mit70 (93.8 Mb) and D11mit214 (114.8 Mb) were intercrossed two times in order to produce the congenic line *Cia40*. All of the mice that were homozygote for *Cia40 *in the study had equal fragment size (Figure 1). However, the heterozygote animals differed slightly in fragment length among the individuals (1 to 2 Mb).

### Genotyping

Genomic DNA was isolated from the tip of the tail according to a previously described protocol [10]. Nine fluorescence-labeled polymorphic microsatellite markers (Interactiva, Biotechnologie GmbH, Ulm, Germany) were used to cover the heterozygous fragment derived from the NFR/N as previously described [10] (Figure 1). The polymerase chain reaction products were analyzed on a MegaBACE™ 1000 (GE Healthcare UK Ltd, Amersham Place, Little Chalfont, Buckinghamshire HP7 9NA, UK) according to the manufacturer's protocol. Data were analyzed with Genetic Profiler 1.1. (GE Healthcare UK Ltd, Amersham Place, Little Chalfont, Buckinghamshire HP7 9NA, UK).

### Induction and evaluation of collagen-induced arthritis

To induce CIA, 8- to 12-week-old mice were immunized subcutaneously at the base of the tail with 100 μg rat collagen type II (CII) emulsified in 0.1 M acetic acid in complete Freund's adjuvant (Difco Laboratories, now part of Becton Dickinson and Company, Franklin Lakes, NJ, USA). After 30 days, a booster injection containing 50 μg CII emulsified in 0.1 M acetum in incomplete Freund's adjuvant (Becton Dickinson and Company) was given. The clinical scoring of arthritis was commenced 25 days after the first immunization. The scoring system is based on the number of inflamed joints, ranging from 1 to 15 for each affected paw. Each affected ankle/wrist was given a score of 5, and each inflamed knuckle and toe was given 1 point. The scores of the four paws were added, yielding a maximum total score of 60 points for each mouse. The severity trait is the maximum score observed in each individual female. Mice that did not develop CIA were given a score of 0 for the traits of severity, onset, and incidence. The onset is the number of days calculated from the first immunization to the first clinical signs of arthritis excluding unaffected animals.

### Enzyme-linked immunosorbent assay

The mice were sacrificed at day 90 and sera were collected. Anti-CII antibody titers in sera were analyzed by a sandwich enzyme-linked immunosorbent assay technique [11]. In short, immunosorbent plates were coated with CII (10 μg/mL) overnight at 4°C. Bovine serum albumin (Sigma-Aldrich, St. Louis, MO, USA) was used for blocking, and thereafter different dilutions of control sera (purified mouse anti-collagen type II antibodies), test sera, and positive and negative controls were added. The presence of CII-specific IgG was visualized by peroxidase-conjugated goat anti-mouse IgG.

### Statistical analysis

Statistical comparison between the different experimental groups was performed by using the Mann-Whitney *U *test.

## Results

### Increased incidence, onset, and severity of collagen-induced arthritis in heterozygous *Cia40 *congenic female mice

**Figure 2:**
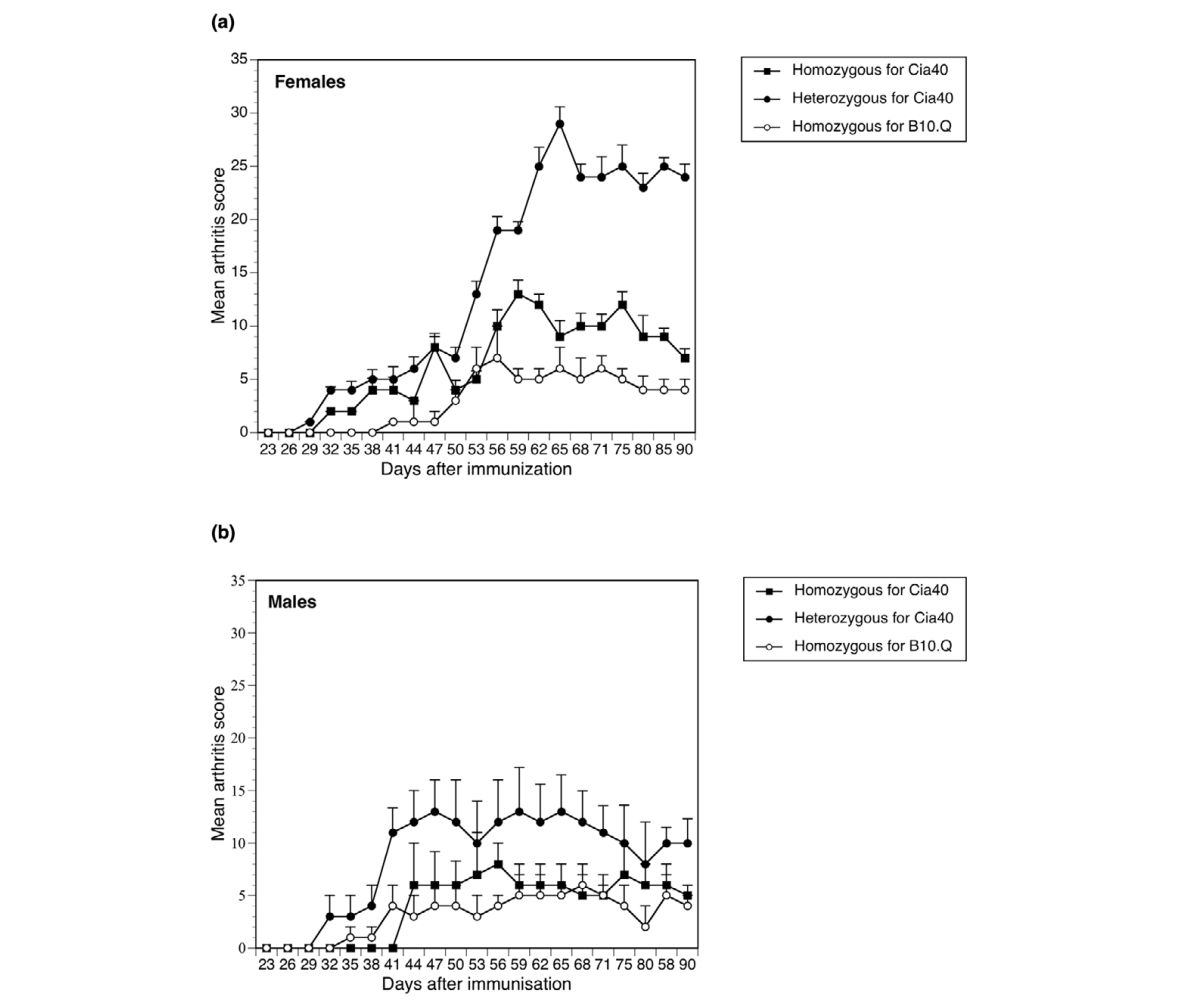
Severity of collagen-induced arthritis in *Cia40 *congenic male and female mice. **(a) **Mean (standard error, SE) arthritic scores in homozygous *Cia40 *congenic females, heterozygous *Cia40 *congenic females, and wildtype littermate females. Only mice that developed arthritis have been included. Heterozygous congenic females show higher severity than wildtype B10.Q and congenic homozygous females (*P *< 0.05). **(b) **Mean (SE) arthritic scores in homozygous *Cia40 *congenic males, heterozygous *Cia40 *congenic males, and wildtype littermate males. Only mice that developed arthritis have been included. Heterozygous congenic males show significantly higher severity than wildtype B10.Q littermates (*P *< 0.05).

Heterozygous and homozygous *Cia40 *congenic mice and corresponding littermate controls of both sexes were immunized with rat CII and monitored three times a week for 90 days. Serum samples for anti-CII antibody analysis were collected at the end point of the experiment. Results presented in Table 1 show that heterozygous *Cia40 *congenic mice suffer from an elevated incidence of the disease. This increase in incidence was particularly obvious and significant in the group of females (*P *< 0.05). Surprisingly, no significant differences in incidence were observed in homozygous *Cia40 *congenic females or males in comparison with the corresponding controls. The onset of the disease was significantly quicker in heterozygous females in comparison with wildtype B10.Q and homozygous congenic littermates. There were no significant differences in onset between the different groups of males. The severity of the disease was elevated in heterozygous *Cia40 *congenic mice of both sexes, as shown in Figures 2a and 2b. Homozygous mice showed a minor increase in severity in comparison with wildtype B10.Q littermates, but this difference was not significant. The heterozygous congenic males showed a higher severity in the beginning of the disease, whereas heterozygous females showed higher severity in the latter part of the disease. The heterozygous congenic females developed a more severe arthritis than the heterozygous congenic male mice. The heterozygous congenic females also showed a significantly shorter onset (*P *< 0.05) of CIA than corresponding controls and all other groups (Table 2).

**Table 1:** Incidence of collagen-induced arthritis in *Cia40 *congenic male and female mice

	Number	Incidence		
		Wildtype B10.Q	Heterozygous *Cia40*	Homozygous *Cia40*
Total	116	12/48 (25%)	24/47 (51%)	12/36 (33%)
Females	54	4/24 (17%)	12/15 (80%)^a^	5/15 (33%)
Males	62	8/24 (33%)	7/17 (41%)	7/21 (33%)

^a^Significantly higher incidence in heterozygous congenic females compared with wildtype littermates (*P *< 0.05).

**Table 2:** Onset of arthritis in *Cia40 *congenic male and female mice

	Number	Onset^a ^(range)		
		Wildtype B10.Q	Heterozygous *Cia40*	Homozygous *Cia40*
Total	116	55 (32, 82)	50 (29, 78)	45 (38, 84)
Females	54	53 (32, 70)	38 (29, 59)^b^	42 (38, 72)
Males	62	59 (35, 82)	56 (32, 78)	52 (47, 84)

^a^Day of onset. Median values for onset calculated on all arthritic mice in the group on day 90. Figures in parenthesis indicate minimum and maximum values for onset. ^b^Significantly shorter onset in heterozygous congenic females compared with wildtype littermates (*P *< 0.05).

### Heterozygous *Cia40/Pregq2 *congenic mice show increased anti-collagen type II antibody levels

Anti-CII antibody titers in serum were analyzed at the end of the experiment (Table 3). The results showed that heterozygous *Cia40 *congenic females develop significantly higher anti-CII antibody titers than wildtype and homozygous congenic mice (*P *< 0.05) of the same sex. No significant differences in anti-CII titers were observed between the different groups of males. This shows that the antibody titers follow the disease phenotype in the congenic mice.

**Table 3:** Anti-collagen type II titers in *Cia40 *congenic male and female mice

	Number	Anti-collagen type II titers at day 90, mg/mL		
		Wildtype B10.Q	Heterozygous *Cia40*	Homozygous *Cia40*
Total	161	0.68 ± 0.24	1.29 ± 0.31	0.86 ± 0.25
Females	54	0.71 ± 0.32	1.57 ± 0.34^a^	0.96 ± 0.24
Males	62	0.67 ± 0.38	0.75 ± 0.21	0.70 ± 0.17

^a^Significantly higher antibody titer in heterozygous congenic females compared with wildtype littermates (*P *< 0.05). Values are presented as mean ± standard error.

### Reduced breeding performance and disturbed breeding behavior in *Cia40/Pregq2 *congenic mice

**Figure 3:**
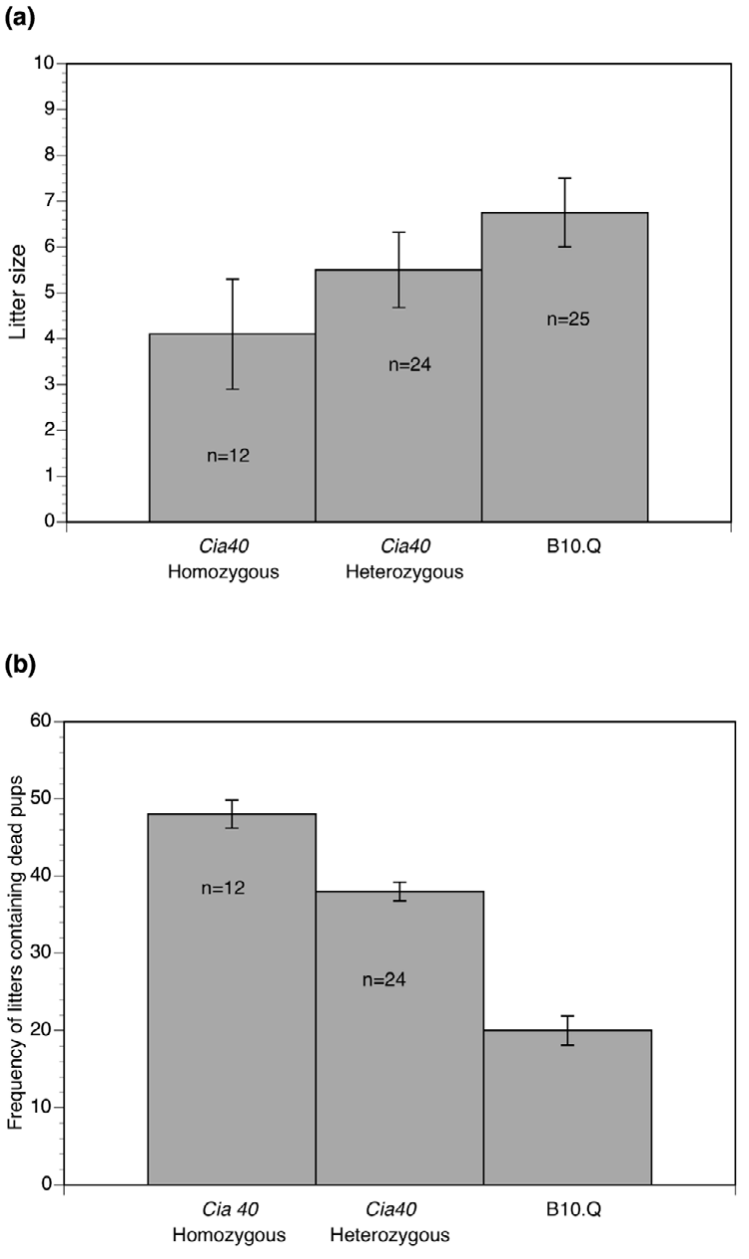
The mean litter size and the frequency of litters containing dead pups in *Cia40 *congenic females and B10.Q wildtype littermates. **(a) **The mean litter size (standard error, SE) in homozygous *Cia40 *congenic females, heterozygous *Cia40 *congenic females, and wildtype B10.Q littermates (n = number of pregnancies). The difference between homozygous *Cia40 *congenic mice and wildtype B10.Q littermates was significant (*P *< 0.05). **(b) **The mean (SE) frequency (percentage) of litters containing dead pups in homozygous *Cia40 *congenic mice, heterozygous *Cia40 *congenic mice, and wildtype B10.Q littermates (n = number of pregnancies). The difference between *Cia40 *homozygous congenics and B10.Q wildtype littermates was significant (*P *< 0.007).

The *Cia40/Pregq2 *congenic mice were difficult to breed and congenic mice of both sexes showed disturbed breeding behavior. Congenic females showed a reduced frequency of successful pregnancies, and pups were frequently killed and eaten shortly after delivery. Figure 3a shows that the mean litter size (surviving pups) of *Cia40 *congenic females crossed with B10.Q males is significantly reduced (*P *= 0.041) compared with the litter size of wildtype littermate females crossed with B10.Q males. Figure 3b shows the frequency of litters containing dead pups (the exact numbers were normally not possible to count) in breeding cages containing *Cia40/Pregq2 *congenic female mice and breeding cages containing only wildtype littermate females. The frequency of litters containing dead pups was dramatically higher in breeding cages containing *Cia40/Pregq2 *congenic females compared with those containing wildtype females (*P *= 0.0069). These data show that the majority of the litters that were born by the congenic females contained nonsurviving pups. The high neonatal mortality among the pups from the congenic females appeared to be due to behavioral disturbance characterized by maternal ignorance and a tendency toward attacking and eating their own pups.

## Discussion

The results of the present study indicate that one or more polymorphic genes in the congenic *Cia40/Pregq2 *fragment affect severity, onset, and incidence of CIA as well as the reproductive performance of B10.Q mice. Interestingly, the increased incidence and severity are pronounced traits in heterozygous mice only, and the influence of the congenic fragment is particularly obvious in the heterozygous females, which actually show a much higher incidence than the males. This is striking since females of the strain B10.Q normally show a very low incidence of arthritis (around 15%). The female predominance in incidence of CIA makes polymorphic genes in the congenic fragment particularly interesting since female predominance is characteristic for RA in humans.

None of the genes close to the calculated position of the *Cia40/Pregq2 *locus is known to be involved in the regulation of inflammation (Table 1). For this reason, we believe that polymorphic or mutated regulatory genes, which in turn affect the activity of several enzymes, could be particularly interesting candidate genes. One such candidate gene is mitogen-activated protein (MAP) kinase, *Map2k6*, which has been reported to affect the function of the immune system. For instance, Ehlting and colleagues [12] recently reported that the regulation of a suppressor of cytokine signalling 3' (SOCS3) mRNA stability by tumor necrosis factor-alpha involves the activation of the MAP kinase cascade. Table 4 shows possible gene candidates, based on single-nucleotide polymorphism data in this particular fragment on chromosome 11 in between inbreed strains of NMRI and C57BL/10 mice from the Wellcome Trust database (gscan) [13].

**Table 4:** Summary of possible candidate genes on chromosome 11 for *Cia40/Pregq2*

Gene	Position, mb	Description	Reproductive or inflammatory phenotypes of mutation
*Ngfr*	95.430132 – 95.449049	Nerve growth factor receptor	Perinatal lethality
*Phb*	95.5528271 – 95.542087	Prohibitin	Lethality before weaning
*Igf2bp1*	95.818477 – 95.867254	Insulin-like growth factor 2	Fetal growth
*Med24*	96.565905 – 98.590749	Mediator complex subunit 24	Pups die prior to birth
*Gsdm3*	98.490658 – 98.499540	Gasdermin	Abnormal loss of skin and hair
*Etv4*	101.631061 – 101.646685	Ets variant gene 4 (E1A enhancer-binding protein, E1AF)	Mammary gland abnormality, male infertility
*Cd79b*^a^	106.172655 – 106.176076	CD79B antigen	Hematopoietic, immune
*Prkar1a*	109.510719 – 109.530970	Protein kinase, cAMP-dependent regulatory, type I, alpha	Embryonic lethality
*Abca8a*^a^	109.886948 – 109.957292	ATP-binding cassette, sub-family A (ABC1), member 8a	Not known
*Map2k6*^a^	110.260436 – 110.386836	Mitogen-activated protein kinase kinase 6	Abnormal immune system
*Sox9*	112.643538 – 112.649074	SRY-box containing gene 9	Perinatal lethality, cartilage formation, sex reversal

^a^Polymorfism between inbreed strains of NMRI and C57BL/10 according to gscan, Wellcome Trust Centre for Human Genetics [13]. Mb for the genes/markers are according to Mouse Ensemble built 36 [18].

We have previously speculated that the same gene(s) might affect both arthritis incidence and pregnancy failure [5]. This assumption is supported by the fact that the incidence of autoimmune CIA is elevated in females but not in males and that the elevated severity is particularly obvious in females. A modified gene that increases the risk of developing autoimmune inflammation in females can also be expected to interfere negatively with pregnancy success. Some types of early pregnancy failures could actually be caused by increased autoimmune reactivity. Again, it is possible that the MAP kinase is involved in the success of implantation. This assumption is strengthened by a recent observation that the MAP kinase cascade indeed affects preimplanted embryos [14]. Still, it might be more likely that different mechanisms and genes are involved in the regulation of arthritic inflammation and the regulation of pregnancy success. If true, this would make it possible to separate *Cia40 *gene(s) from the breeding-suppressing *Pregq2 *gene(s), which would be of great advantage for the future characterization of the part of the *Cia40 *gene(s) that influence the outcome of arthritis.

The observation that the heterozygous *Cia40 *congenic mice show a quicker onset, and in the case of males, also develop a more severe disease, raises questions about the molecular mechanisms controlling arthritis. A polymorphism leading to an amino acid substitution in one allele could have strong effects on the function of a di- or multimeric protein and polymorphisms in noncoding regulatory regions could result in skewed transcription and altered protein levels. The observed phenotypic effects due to heterozygous alleles might be helpful in the identification of candidate genes. The heterozygous effect has previously been reported in a study of CIA development, in which mice with heterozygous alleles in a congenic fragment on mouse chromosome 15 were much more affected by the disease than homozygous littermates were [15].

We have found only a limited number of genes in the vicinity of the *Cia40 *and *Pregq2 *peaks, which show polymorphism between B10 and NMRI. In addition to *Mapk6*, we have focused some attention on the *Abca8a *gene and *CD79b *gene. The role of the *Abca8a *gene in the context of reproduction and immunity is largely unknown, whereas the *CD79b *gene is of importance primarily in the context of B-cell development [16]. At present, it is not possible to speculate about the possible influence of these two genes for the phenotypes observed, but the function of these genes does not make them our main candidate genes.

The interesting reversal of sex susceptibility to arthritis and the observations that congenic males show impaired development of genital organs and that females are more aggressive and less caring mothers have made us pay attention to the *Sox9 *gene. The *Sox9 *gene has been reported to cause sex reversal [17], which is a highly relevant phenotype in the context of the *Cia40/Pregq2 *congenic mice. The possible presence of a *Sox9 *polymorphism/mutation on chromosome 11 in our congenic mice is under investigation.

## Conclusion

The present results show that the *Cia40 *locus on chromosome 11 contains one or more polymorphic genes that particularly influence incidence and severity of CIA in female mice. These effects are significant in congenic B10.Q female mice carrying heterozygous *Cia40 *fragments of NFR/N origin. Congenic mice carrying heterozygous fragments also show quicker onset of the disease. The major polymorphic candidate genes in the congenic fragment are *Cd79b*, *Abca8a*, and *Map2k6*. The NFR/N fragment present in the congenic mice also contains a locus denoted *Pregq2*, which causes a change in reproductive behavior and reduces pregnancy success. This effect is significant in congenic B10.Q females carrying a homozygous NFR/N fragment. The *Sox9 *gene, known to influence sex reversal, is a candidate gene for the reproductive phenotype.

## Abbreviations

CIA = collagen-induced arthritis; CII = collagen type II; EAE = experimental autoimmune encephalitis; MAP = mitogen-activated protein; QTL = quantitative trait locus; RA = rheumatoid arthritis.

## Competing interests

The authors declare that they have no competing interests.

## Authors' contributions

ML was responsible for genotyping, phenotyping, and analysis and helped to interpret the data and write the manuscript. RM, ÅA, and RH helped to interpret the data and write the manuscript. All authors read and approved the final manuscript.
